# Supplementary material for: Single-molecule imaging reveals replication fork coupled formation of G-quadruplex structures hinders local replication stress signaling
Source: Nat Commun. 2021 May 5;12:2525. doi: 10.1038/s41467-021-22830-9 (PMC8099879; doi:10.1038/s41467-021-22830-9)
Supplement: Supplementary file 1 — Supplementary Information [file 41467_2021_22830_MOESM1_ESM.pdf]

Supplementary Materials for

Single-Molecule Imaging Reveals Replication Fork Coupled Formation of G-quadruplex Structures Hinders Local Replication Stress Signaling

Wei Ting C. Lee[1], Yandong Yin[1], Michael J. Morten[1], Peter Tonzi[1], Pam Pam Gwo[1], Diana C. Odermatt[2], Mauro Modesti[3], Sharon B. Cantor[4], Kerstin Gari[2,5], Tony T. Huang[1], Eli Rothenberg[1]*.

Correspondence to: Eli.Rothenberg@nyumc.org.

This file includes:

Supplementary Figures 1 to 5
Supplementary Tables 1 to 3
Supplementary Notes 1 to 3
Supplementary References

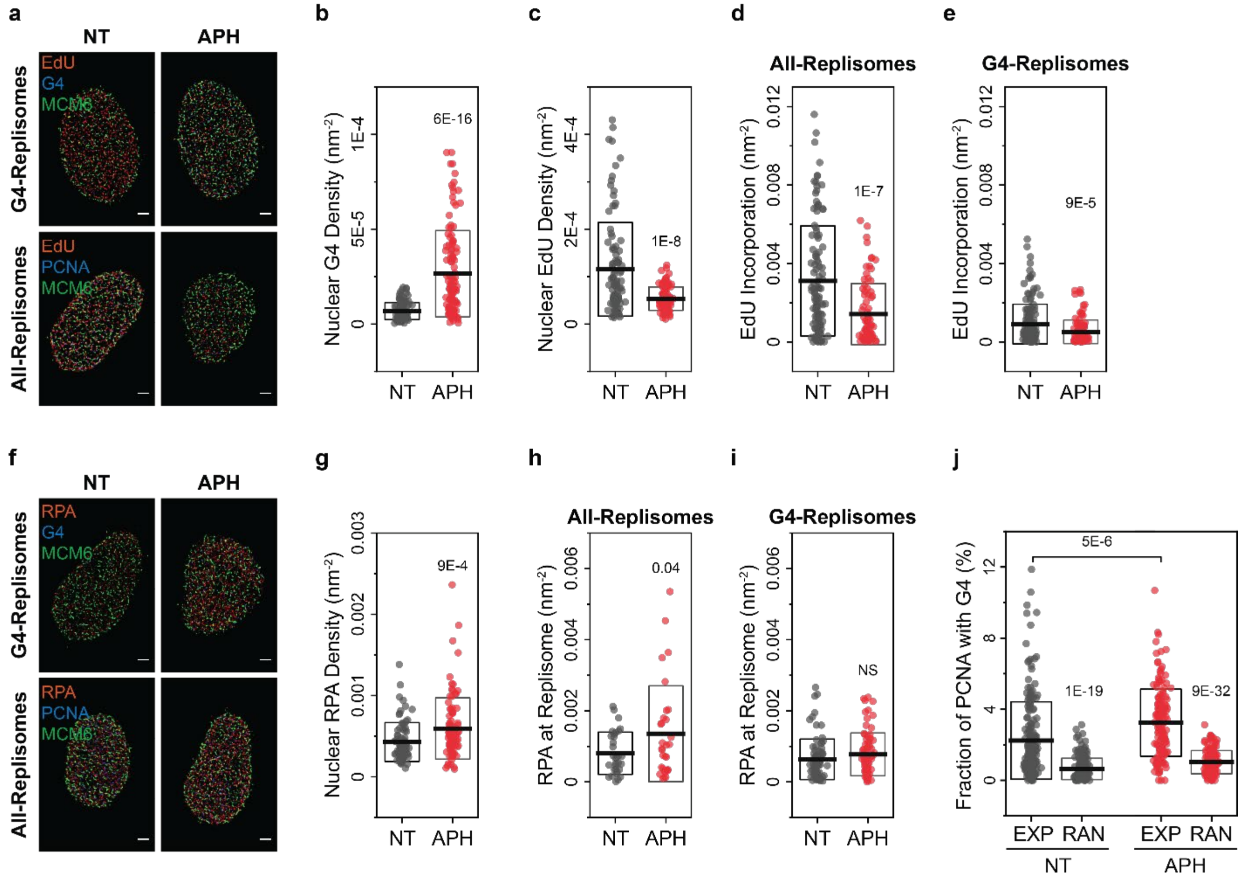

Supplementary Figure 1. SMLM visualization and quantification of DNA G4s and their spatial associations with replisome upon APH treatment.

(**a** and **f**) Representative SMLM images of NT or 1hr, 200nM APH-treated S-phase U2OS nuclei labeled for the indicated targets. Top, G4 Replisomes; bottom, All Replisomes. Scale bars, 2 μm. (**b, c, g**) Nuclear densities of G4 (b), EdU (c), or RPA (g) in NT or APH-treated cells, calculated with AC analysis. (**d, e, h, i**) Local densities of EdU (d and e) or RPA (h and i) at All-Replisomes or G4-Replisomes in NT or APH-treated cells, calculated with TC analysis. (**j**) Fraction of PCNA foci that is associated with G4 foci in NT or APH-treated cells, calculated with DBSCAN/NND analysis. The colocalizations between PCNA and G4 from experiment (EXP) and randomized (RAN) samples are compared to show the significance of colocalization. For (b-e, g-j), individual data points represent result from single cell. Black horizontal line and box height indicate mean±SD. Values on graph indicate *p*-values of unpaired two-sample t-tests. For all experiments, number of cells analyzed and TC triplets identified are listed in Supplementary Table 1.

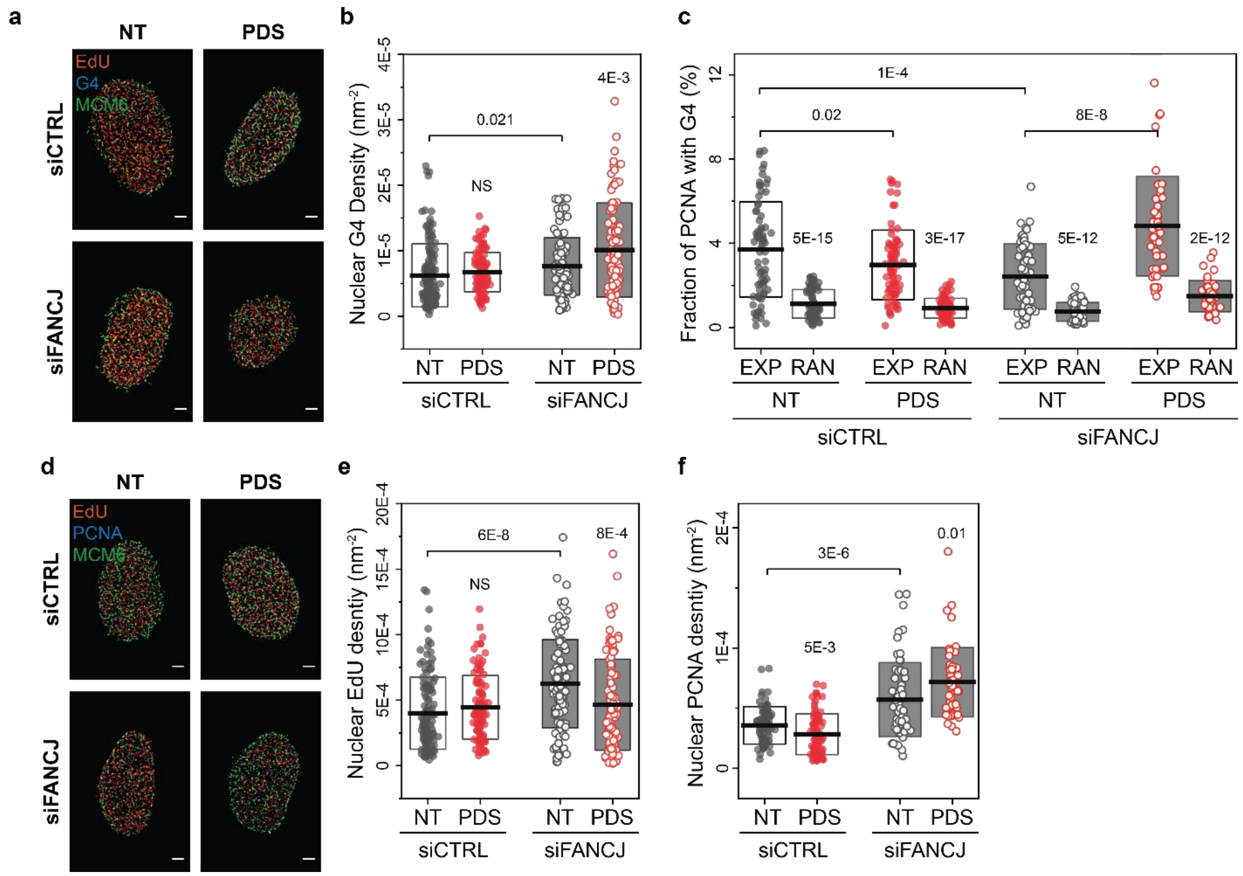

Supplementary Figure 2. SMLM visualization and quantification of DNA G4 structures and their spatial associations with replisome upon PDS treatments.
(**a** and **d**) Representative SMLM images of NT or 1hr, 20μM PDS-treated siCTRL or siFANCJ U2OS nuclei labeled for the indicated targets. Scale bars, 2 μm. (**b, e, f**) Nuclear densities of G4 (b), EdU (e), or PCNA (f) in NT or PDS-treated siCTRL or siFANCJ cells, calculated with AC analysis. (**c**) Fraction of PCNA foci that is associated with G4 foci in NT or PDS-treated siCTRL or siFANCJ cells, calculated with DBSCAN/NND analysis. The colocalizations between PCNA and G4 from experiment (EXP) and randomized (RAN) samples are compared to show the significance of colocalization. Noted that the fraction of PCNA associated with G4 is lower in NT siFANCJ cells than in siCTRL cells. We reasoned that this is due to the increased amount of overall abundance of active replication forks in when FANCJ is depleted [1]. The level of active replication was quantified via AC analysis of EdU (e) and of chromatin bound PCNA (f), with each serving as distinct marker of active replications forks. This revealed a ~55-60% increase in the active replication level in NT-siFANCJ cells as compared to siCTRL cells, supporting that extra origin firing results in the relative decrease in G4-replication fraction in siFANCJ cells. For (b, c, e, f), individual data points represent result from single cell. Black horizontal line and box height indicate mean±SD. Values on graph indicate *p*-values of unpaired two-sample t-tests. For all experiments, number of cells analyzed are listed in Supplementary Table 1.

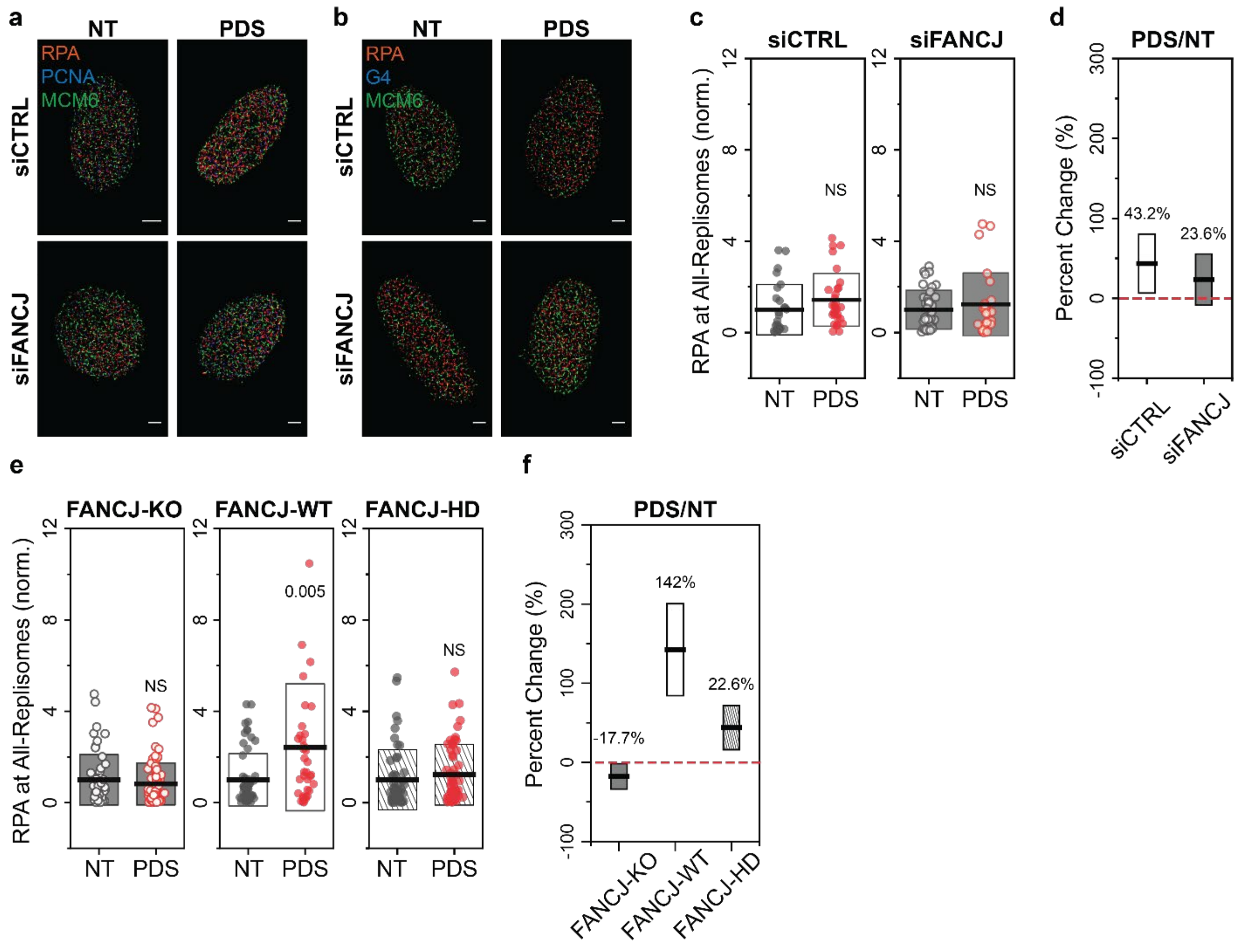

Supplementary Figure 3. Upon G4 stabilization, FANCJ-deficient cells resist RPA chromatin loading.

(**a** and **b**) Representative SMLM images of NT or 4hr, 20μM PDS-treated siCTRL or siFANCJ U2OS labeled for the indicated targets. Scale bars, 2 μm. (**c**) Local densities of RPA at All-Replisomes in NT or 1hr PDS-treated siCTRL or siFANCJ U2OS cells. We did not observe any significant changes in the levels of RPA recruitment under these conditions in either cell-lines, indicative of rapid cellular response in resolving stable G4s shortly after PDS treatment [2]. Based on this, we projected that longer durations of PDS treatments were needed to reveal such changes. We therefore extended the duration of PDS treatment and found that 4-hr treatment was sufficient to induce an increase in RPA signal at All-Replisomes in siCTRL cells (Figs. 4). (**e**) Local densities of RPA at All-Replisomes in FANCJ-knock-out HeLa cells (FANCJ-KO) complemented with wild-type (FANCJ-WT) or helicase-dead (FANCJ-HD) FANCJ. For (c and e), individual data points represent result from single cell. Black horizontal line and box height indicate mean±SD. Values on graph indicate *p*-values of unpaired two-sample t-tests between NT and PDS-treated cells. (**d** and **f**) Percent change of the densities of RPA at All-Replisomes in PDS-treated compared to NT cells. Values on the graph and black horizontal line represent the respective percent changes, box height indicates the propagated s.e.m.. For all experiments, number of cells analyzed are listed in Supplementary Table 1.

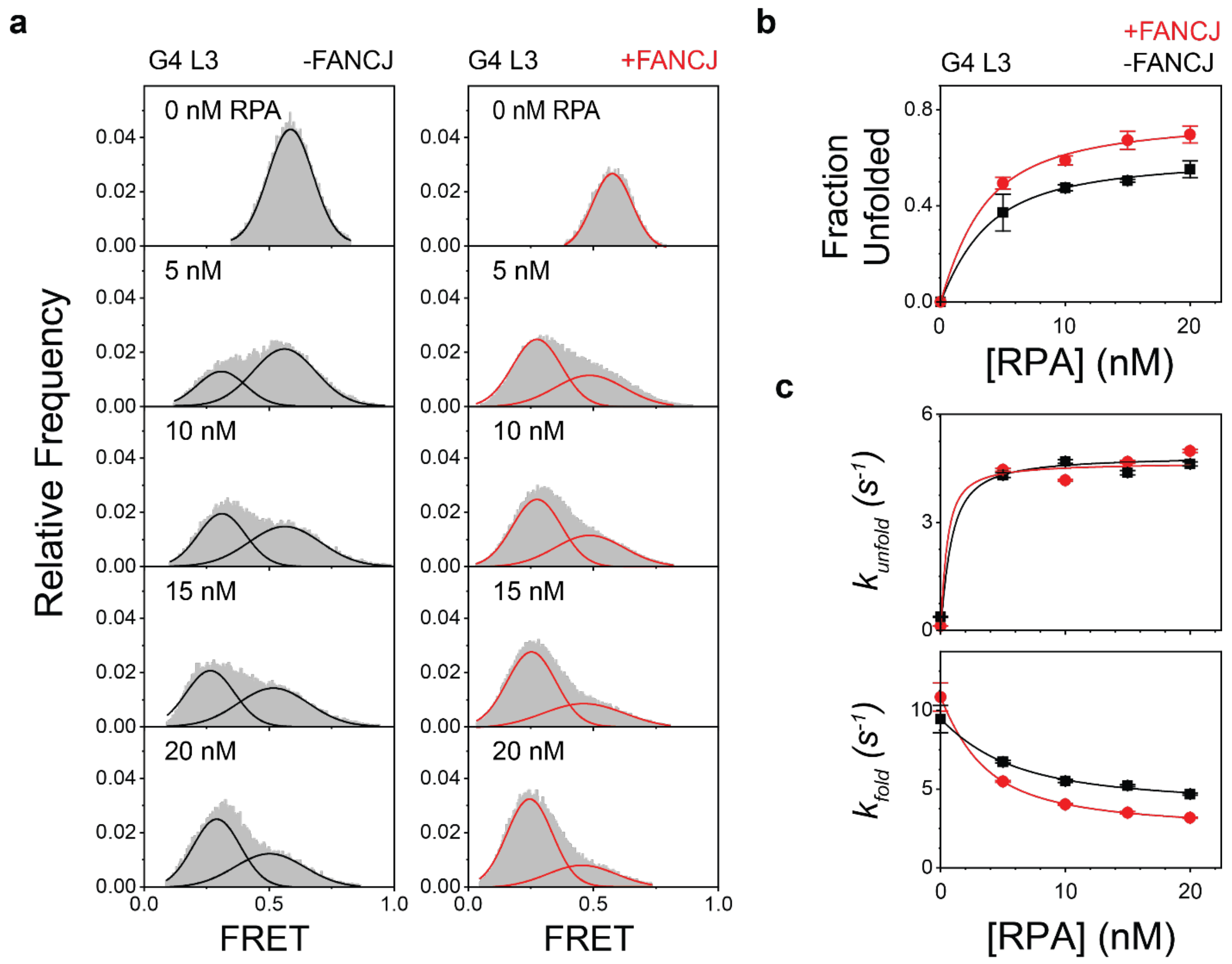

Supplementary Figure 4. Mechanistic insights on FANCJ and RPA during G4 resolution using smFRET.

(**a**) FRET histograms of L3 G4 in the presence of indicated concentrations of RPA with either no (left panel) or 100 pM FANCJ (right panel). (**b**) Fractions of unfolded L3 G4 monitored by smFRET as a function of RPA concentration with (red) or without (black) the addition of FANCJ. Error bars represent mean ± s.e.m.. (**c**) Unfolding (top) and folding (bottom) rates for L3 G4 as a function of RPA concentration with (red) or without (black) the addition of FANCJ. Error bar represents the SD of exponential fit. For all smFRET experiments, a minimum of 100 smFRET trajectories from two independent experiments were used for analysis.

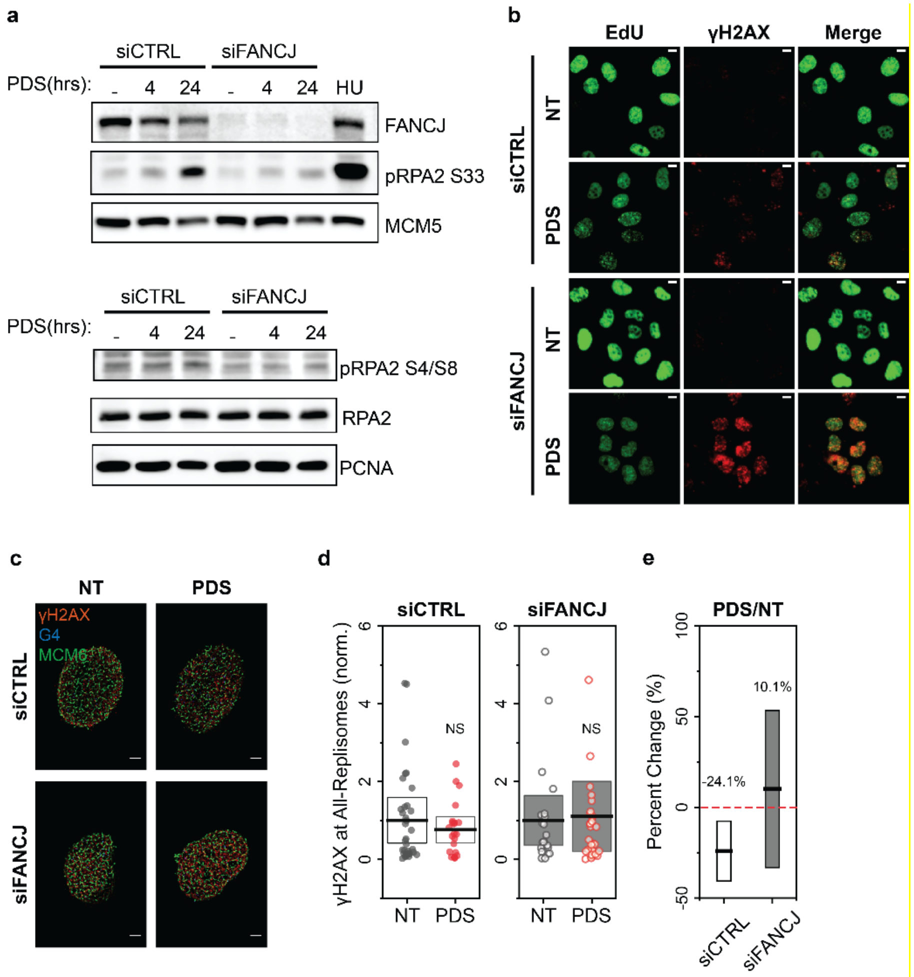

Supplementary Figure 5. FANCJ deficiency leads to reduced RPA signaling and increased DNA damage upon G4 stabilization.

(**a**) Western blots of pRPA2 S33 or S4/S8 in siCTRL or siFANCJ U2OS cells with or without PDS treatments, or siCTRL cells treated with HU as a positive control. Staining of FANCJ confirms siRNA knockdown efficiency. Staining for MCM5 and RPA2 act as controls. Experiments were performed in duplicate. The uncropped blots are provided in Source Data. (**b**) Representative immunofluorescence images of NT or 4hr, 20μM PDS-treated siCTRL or siFANCJ U2OS cells stained with EdU (green) and γH2AX (red), and the overlay of the two images are shown. Scale bars, 10 μm. At least 300 EdU-positive cells were imaged. (**c**) Representative SMLM images of NT or PDS-treated siCTRL or siFANCJ U2OS nuclei labeled for the indicated targets. Scale bars, 2 μm. (**d**) Local densities of γH2AX at All-Replisomes in NT or PDS-treated siCTRL or siFANCJ cells. Individual data points represent result from single cell. Black horizontal line and box height indicate mean±SD. Values on graph indicate *p*-values of unpaired two-sample t-tests between NT and PDS-treated cells. (**e**) Percent change of the densities of γH2AX at All-Replisomes in siCTRL or siFANCJ PDS-treated compared to NT cells. Values on the graph and black horizontal line represent the respective percent changes, box height indicates the propagated s.e.m.. For (d and e), number of cells analyzed are listed in Supplementary Table 1.

Related to	SMLM of	Local Density of	FANCJ knockout	Drug	µM	ROI Analyzed	TC Triplets
Fig. 1 Figure S1	EdU/G4/MCM (G4-Replisomes)	G4		APH	0	244	125
					0.2	218	93
Fig. 2 Fig. S1	RPA/PCNA/MCM (All Replisomes)	RPA		APH	0	80	32
					0.2	95	31
Fig. 2 Fig. S1	RPA/G4/MCM (G4-Replisomes)	RPA		APH	0	80	81
					0.2	95	76
Fig. 2 Fig. S1	EdU/PCNA/MCM (All Replisomes)	EdU		APH	0	244	115
					0.2	218	82
Fig. 2 Fig. S1	EdU/G4/MCM (G4-Replisomes)	EdU		APH	0	244	155
					0.2	218	115
Fig. 3 Fig. S2	EdU/G4/MCM (G4-Replisomes)	G4	siCTRL	PDS	0	156	55
					20	135	72
			siFANCJ		0	112	44
					20	99	55
Fig. 3 Fig. S2	EdU/PCNA/MCM (All Replisomes)	EdU	siCTRL	PDS	0	156	53
					20	135	47
			siFANCJ		0	112	47
					20	99	46
Fig. 3 Fig. S2	EdU/G4/MCM (G4-Replisomes)	EdU	siCTRL	PDS	0	156	82
					20	135	83
			siFANCJ		0	112	64
					20	99	55
Fig. 4 Fig. S3	RPA/PCNA/MCM (All Replisomes)	RPA	siCTRL	PDS	0	103	27
					20-1h	130	30
					20-4h	121	34
			siFANCJ		0	108	38
					20-1h	111	26
					20-4h	99	33
Fig. S3	RPA/PCNA/MCM (All Replisomes) HeLa	RPA	FANCJ -/- + WT	PDS	0	140	61
					20	111	37
			FANCJ -/-		0	80	53
					20	113	84
			FANCJ -/- + HD		0	62	52
					20	72	60
Fig. 4 Fig. S3	RPA/G4/MCM (G4-Replisomes)	RPA	siCTRL	PDS	0	95	66
					20	99	63

			siFANCJ		0	92	59
					20	105	57
Fig. 6 Fig. S5	γH2AX/G4/MCM (G4-Replisomes)	γH2AX	siCTRL	PDS	0	86	56
					20	72	46
			siFANCJ		0	73	54
					20	68	39
Fig. S5	γH2AX/G4/MCM (All-Replisomes)	γH2AX	siCTRL	PDS	0	86	34
					20	72	22
			siFANCJ		0	73	24
					20	68	31

Supplementary Table 1. Number of cells analyzed and TC Triplets resolved in SMLM studies.

Target	Species/ Conjugate	Manufacturer	Produce Code	Application	Dilutions (1º/2º)
DNA G4 (1H6)	Mouse	Millipore	MABE1126	IF	1:200/1:5000
DNA G4 (1H6)	Mouse	Absolute Antibody	Ab00389-1.1	IF	1:200/1:5000
DNA G4 (1H6)	Rabbit	Absolute Antibody	Ab00389-23.0	IF	1:200/1:5000
DNA G4 (BG4)	FLAG-tag	Millipore	MABE917	IF	1:200/1:5000
MCM6	Rabbit, AF568	Abcam	ab211916	IF	1:1000
PCNA	Mouse	SCBT	sc56	IF	1:1000/1:5000
γH2AX	Mouse, AF647	Millipore	05-636-AF647	IF	1:10000
RPA1	Rabbit, AF647	Abcam	ab199240	IF	1:5000
Rabbit IgG	AF 750	Invitrogen	A21039	IF	See above
Mouse IgG	AF 750	Invitrogen	A21037	IF	See above
Rabbit IgG	AF 647	Invitrogen	A21246	IF	See above
Mouse IgG	AF 647	Invitrogen	A21235	IF	See above
Rabbit IgG	AF 568	Invitrogen	A11036	IF	See above
Mouse IgG	AF 568	Invitrogen	A11031	IF	See above
Mouse IgG	AF 488	Invitrogen	A11029	IF	See above
Rabbit IgG	Biotin	Abcam	Ab6720	SimPull	See above
RPA2	Rabbit	Bethyl	A300-244A	WB	1:5000
pRPA2 S4/S8	Rabbit	Bethyl	A700-009	WB	1:5000
pRPA2 S33	Rabbit	Bethyl	A300-246A	WB	1:5000
FANCJ	Rabbit	Bethyl	A300-561A	WB	1:5000
MCM5	Rabbit	Bethyl	A300-195A	WB	1:5000
PCNA	Mouse	Abcam	Ab29	WB	1:5000

Supplementary Table 2. Antibodies used in this study.

Name	5’-3’ Sequence	Used in
Biotin-duplex top	/5Cy5/GCC TCG CTG CCG TCG CCA /3BioTEG/	SimPull / smFRET
Telomeric G4	TGG CGA CGG CAG CGA GGC /iCy3/GGG TTA GGG TTA GGG TTA GGG TTT TTT TTT TTT TTT TTT TT	SimPull
$(dT)_{20}$	TGG CGA CGG CAG CGA GGC TTT TTT TTT TTT TTT TTT TT/iCy3/	SimPull
1nt loop G4 w/ 10nt spacer	TGG CGA CGG CAG CGA GGC TTT TTT TTT TGG GTG GGT GGG TGG G/iCy3/TT TTT TTT TTT TTT TTT TTT	smFRET (L1)
Telomeric G4 w/ 10nt spacer	TGG CGA CGG CAG CGA GGC TTT TTT TTT TGG GTT AGG GTT AGG GTT AGG G/iCy3/TT TTT TTT TTT TTT TTT TTT	smFRET (L3)

Supplementary Table 3. DNA Substrates used in this study.

Supplementary Note 1: Validation of the G4 antibodies.

To image DNA G4 structures in SMLM, we tested and further optimized the two previously validated G4-specific antibodies, 1H6 [3] and BG4 [4], confirming their activities. Cells that were co-stained with 1H6 and BG4 displayed significant overlaps between the two antibodies (Supplementary Note 1 Fig. 1a), indicating that both antibodies predominantly recognize G4 structures in cells. We further quantified their colocalizations by comparing the magnitude of their colocalizations within the nuclei with that of simulated images portraying uncorrelated relationship between the two antibodies, in which random colocalization is expected, further establishing an overlap in their specificities (Supplementary Note 1 Fig. 1b). Since BG4 was found to target a broader range of G4 structures, including RNA G4s [5], we have decided to predominantly use 1H6 in our SMLM experiments to lower possible backgrounds in our imaging. The single-molecule specificity of 1H6 was also validated by performing modified single-molecule pulldown assays (SimPull) [6]. These measurements confirmed the selective binding of 1H6 to folded DNA G4 substrates, but not to random ssDNA substrates (Supplementary Note 1 Fig. 1d-e).

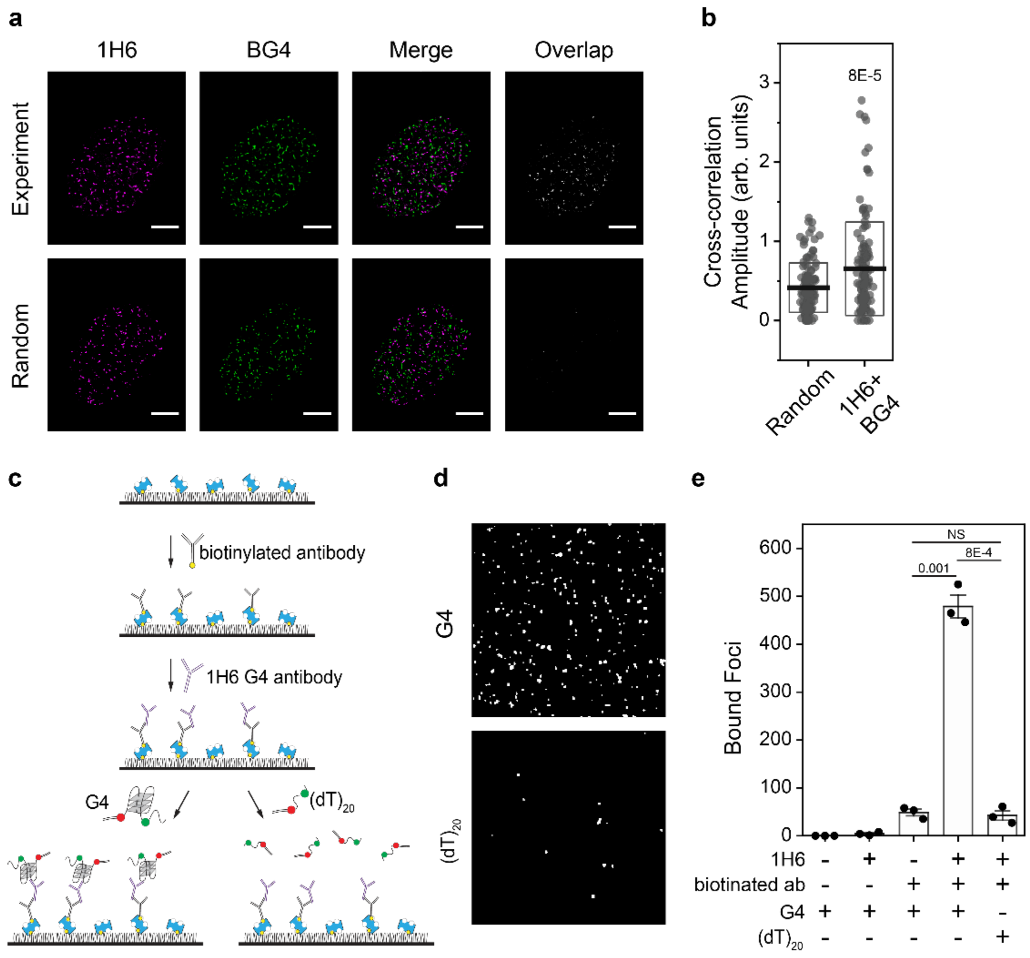

Supplementary Note 1 Figure 1. Validations of 1H6 G4 antibody.

(**a**) 'Experiment': Representative SMLM images of U2OS nucleus labeled with 1H6 (Magenta) or BG4 (Green). 'Overlap' shows the areas where the two colors overlap. 'Random': All 1H6 foci from the nucleus were shuffled to visualize the level of random overlap between the two colors. Scale bars, 2 μm. (**b**) Cross-Correlation analysis showing the magnitude of colocalization between 1H6 and BG4 from experiment and randomized samples. 1H6 and BG4 significantly colocalizes to each other as compared to random distribution. Black horizontal line and box height indicate mean±SD. N = 125. Values on graph indicate p-values of unpaired two-sample t-tests against randomized data. (**c**) Schematic illustration for the SiMPull assay. Microscope slides and coverslips are passivated with biotin-PEG. Biotinylated secondary antibodies against the 1H6 antibody are immobilized to the bound NeutrAvidin (blue object). 1H6 antibody is then immobilized by binding to the biotinylated antibodies. When fluorescent-labeled DNA G4 substrate is added to the chamber, the surface immobilized antibody captures the substrate. $(dT)_{20}$ oligos on the other hand do not bind and are washed away. (**d**) Representative images showing the capture of fluorescently labeled DNA G4 (top) or $(dT)_{20}$ (bottom). (**e**) Quantification of bound molecules upon the addition of different components. Data collected from three experiments, shown as black datapoints, were pooled together for analysis. Error bars represent mean ± s.e.m.. Values on graph indicate p-values of unpaired two-sample t-tests.

Supplementary Note 2: Estimating the fraction of replication sites with G4 signals. Our multi-color SMLM platform has enabled us to determine the association of DNA G4 structures with the replication machinery. By utilizing quantitative SMLM clustering (DBSCAN) and colocalization (Nearest Neighbor Distance (NND)) approaches [7, 8], we estimated ~2.24% of the observed replication sites form G4 structures. To determine the robustness of this quantification, we estimated the percent-range of G4-positive RFs that are expected to be observed in a single cell, derived from computational predictions of genomic G4 motifs, genomic data, and live-cell imaging studies:

- **Estimates based on genomic data:** In terms of the prevalence of G4-containing sequence, genomic analyses provide an estimate of approximately 700,000 G4 motifs with elaborate structural considerations [9, 10]. However, given the sequence variability and thermodynamic stability of G4s, as well as the complexity of cellular environment, most of these motifs are unlikely to form G4 structures in the cell, and when formed these can be readily destabilized by DNA binding proteins such as RPA. Of the overall population of G4 motifs, it was found that there is an increase representation for G4 motifs having short loops. Specifically, in the human genome, single-nucleotide loop motifs (G4-L1) are statistically overrepresented and make up ~5% of all G4 motifs [9]. Of the total G4-L1 motifs, the thermal stabilities (experimental $T_{1/2}$, derived by their melting curves) of G4-L1A (A-A-A loops) and mixed-base loops are not likely to pose an issue as they can be destabilized by RPA, whereas G4-L1T, G4-L1G and G4-L1C (T-T-T, G-G-G, and C-C-C loops) are more stable ($60\ ^{O}C < T_{1/2} < 65\ ^{O}C$) and therefore can contribute to genomic instability, as shown by the Nicolas lab [11]. While the total number of G4-L1 motifs

in the human genome is estimated to be close to 39,000, the more thermodynamically stable G4-L1 motifs (G4-L1T, G4-L1G and G4-L1C) with higher chance of stably forming into a G4 in cell are only ~9% of that population, which is approximately ~3,500 motifs [9]. Considering the genome size to be ~ 6 billion base pairs, with the average size of a replicon to be 100-120kb [12], each RF is about 50-60kb, and ~100,000 forks in total are needed to replicate the entire genome in one cell. Based on this, we estimate that a total of **3.5%** (3,500 G4 motifs/100,000 forks) of forks could 'encounter' a stable G4-L1 motifs throughout the course of the entire S phase. The actual fraction would vary from early to late S-phase with changes in replication pace, distribution and whether the stable G4-L1 motifs actually fold into G4 structures. Thus, the percentage of observed forks with G4 structures is expected to be in the range of 2-3%.

- **Estimates based on G4-ChIP-seq measurements**: A recent study using G4-ChIP-seq had detected ~1,000 to ~10,000 G4 in different human cell-lines [13]. Using these values, we estimate the probability of observing a G4-RF to be 1-10%.
- **Estimates based on live cell studies**: In a very recent paper utilizing elegant detection of G4s in live cells, the authors have had estimated a total number of G4s in a single U2OS cell of ~3000 [14]. Using this value, the fraction of G4-RF would be ~3%.

While our observations (~2.24%) is consistent with the estimates provided above, we would like to emphasize that our calculations are only mean to provide an approximation rather than an absolute number. In this manuscript we also utilized the probabilistic TC analysis approach (Supplementary Note 3)[15, 16] to measure the correlation between G4

and replisome complex (MCM-EdU). We found that in intact cells, the three components significantly associate with each other. This provides, to the best of our knowledge, the first direct quantitative visualization of G4 localizing within the replisome, addressing the previously unanswered question of how G4 can interact with and affect the replisome.

Supplementary Note 3: Principle of Triple-Correlation Function.

Multi-color single-molecule localization microscopy (SMLM) is one of the leading super-resolution imaging approaches that enables for visualization of spatial organizations of molecular complexes at nanoscale level inside cells [17, 18]. However, as we obtained more detailed information from the highly sensitive SMLM imaging, we also face challenges in accessing the numerous information resolved from SMLM images. This is especially critical for analyzing images with particularly dense populations (such as the highly abundant replication proteins inside a nucleus) that are subjected to heterogeneous distributions, various orientations, and random co-localization incidence. To address these challenges, we utilized an algorithm employing the Triple-Correlation (TC) Function for unbiased pattern recognition and quantification of any three-component-labeled molecular complexes in three-color SMLM images [15, 16].

The TC algorithm samples the triplet complexes configured by all molecules from each of the three labeled species and computes their probability density as a function of their relative displacement. In brief, the TC algorithm samples every possible three-component geometric pattern formed by any of the individual red, green, and blue signals from a nucleus (Supplementary Note 3 Fig. 1a and b). If a triplet pattern, defined by the length of the edges of the triplet, constantly presents within the nucleus, it can be identified by the TC analysis as a TC triplet as its population distinguishes from other stochastic combination of the red, green, and blue molecules (Supplementary Note 3 Fig. 1c), whereas if the distributions of the three colors are not correlated with each other (i.e. random distribution), no TC triplet will be identified [15, 16]. By overlaying different TC triplets acquired from multiple nuclei we imaged and analyzed, such that the relative frequencies

of the TC triplets from each nucleus are represented by the sizes of the circles, we then obtain a statistical description of the spatial relationship among the three colors (Supplementary Note 3 Fig. 1d and e). We note that when the scale of the pattern is comparable to or smaller than the combined spatial resolution (~40 nm), the pattern tends to present as an equilateral triangles due to the disparity between the center-to-center distance and the average distance among three co-localized clusters that can overlap with each other (see Supplementary Figure 5 in [16]). This, however, does not affect the quantifications of the components within each pattern, as discussed below.

Besides the three-component geometric pattern(s), the algorithm allows for the estimations of molecular content within the TC triplet as it computes the averaged probability density of the molecules within the TC triplet (see method, Supplementary Note 3 Fig. 2). Note that throughout this work, this information is displayed on the circle sizes of the TC triplets – enlarging circle size means increasing local density of the molecule of interest.

A significant advantage of the TC analysis is that it can distinguish the presence of a specific molecule-of-interest in different molecular complexes. For example, in Supplemental Note 3 Figure 3a we simulated a series of four-color SMLM images with notably colocalizing Blue-Red-Green (B-R-G) and Yellow-Blue-Red-Green (Y-B-R-G) complexes. Note that the local densities of RED only increases within the Y-B-R-G complexes but not within the B-R-G complexes. As we submit these simulations for TC computation to quantify the relative local densities of RED, we found a significant increase trend for RED within the Y-R-G triplets (Supplementary Note 3 Fig. 3b) but a mild increase trend for RED within the B-R-G triplets (Supplementary Note 3 Fig. 3c). On the other

hand, as we simulate a series of images with increasing RED within the B-R-G complexes but not the Y-B-R-G complexes (Supplementary Note 3 Fig. 3d), we did see a significant increase trend for RED only within the B-R-G triplets (Supplementary Note 3 Fig. 3f), but not within the Y-R-G triplets (Supplementary Note 3 Fig. 3e). We used this strategy in the main text to distinguish the local abundances of RPA and EdU at progressing replisomes with or without G4 associations.

As we couple SMLM imaging with TC analysis, we note that despite extensive sample optimization (detailed below) the antibody labeling efficiency for each targeted molecule, as well as the various photoswitching properties of different fluorophores, may still affect the number of detected molecules of each target with respect to their actual amounts in cells, and consequently the correlation frequency among the three coupled molecules. To address these issues, we analyzed the triple-correlation of EdU+PCNA (both served as replication fork markers, Supplementary Note 3 Fig. 4a) or RPA (Supplementary Note 3 Fig. 4b) were triple-stained in different colors. We found their resulting triple-correlation magnitude, regardless of the color combination, to be significantly stronger than the correlation magnitude obtained from simulated images with random colocalizations (see method and Fig. S3 and Fig. S4 in Chen, Y. H. *et al.*[19] for randomization procedure). These measurements indicate that the TC approach is capable of robust determination of distinct molecular configurations despite potential under-sampling due to different fluorophore photoswitching properties, labeling efficiencies, or the crowded environment of our region of interest (the nucleus). It is important to note that the distinct configurations are well resolved notwithstanding the expected changes in the absolute TC magnitudes obtain for different color combinations. Finally, we note that in order to minimize errors

and variabilities that may arise from different blinking and labeling biases we thoroughly test, validate and optimize the labeling conditions for each molecule-of-interest. Once these conditions are established, we maintain these conditions throughout our experiments by using the same antibody dilutions and fluorophore conjugations for the same targets to avoid any comparison of the same target between different antibodies and different colors (Supplementary Table 2).

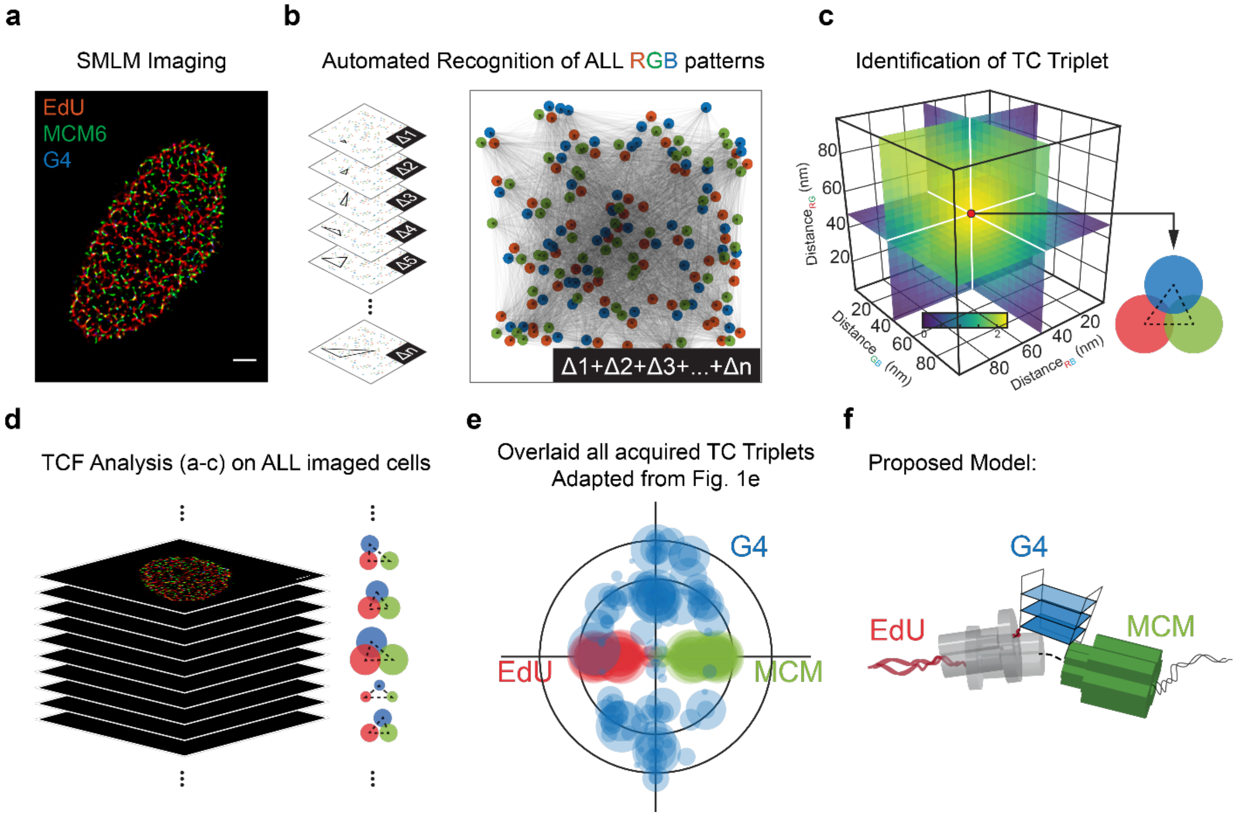

Supplementary Note 3 Figure 1. Step-by-step illustration of TCF Analysis.

(**a**) SMLM Imaging. Representative SMLM image of a U2OS nucleus labeled with EdU (red), MCM (green), and G4 (blue). Scale bar: 2 μM. (**b**) Automated recognition of all RGB patterns. As we submit the coordinates of Red, Green, Blue from (a) to TC analysis, the algorithm will identify all possible Red-Green-Blue triplet configurations (Δ1 to Δn), and each configuration is defined by the length of its three sides. (**c**) Identification of TC Triplet. Left: 3-D heatmap (Equation 1) reflecting the frequency of each configuration recognized in (b) as a function of the lengths of their three sides ($Distance_{RG}$, $Distance_{RB}$, $Distance_{GB}$). Right: TC Triplet resolved from (a); circle sizes represent the average density of G4 (Blue) at each TC triplet within the nucleus. (**d** to **e**) TC Analysis of all imaged cells. Steps (a-c) are repeated for every imaged nucleus, generating multiple TC Triplets (d). All resolved TC triplets are overlaid together, reflecting a statistic description of the association pattern among EdU, MCM, and G4 (e). (**f)** Proposed model based on TC result from (e).

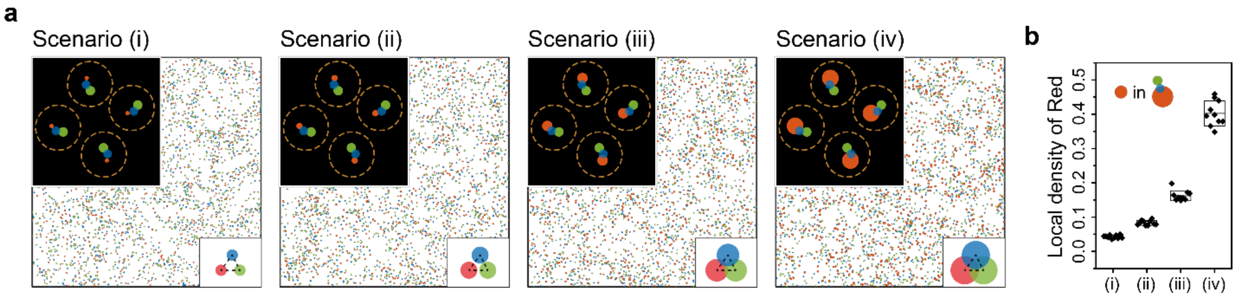

Supplementary Note 3 Figure 2. Validation of the TCF-resolved local density within a triplet pattern via simulations.

(**a**) 1000 RGB triplets were randomly positioned on the canvas, where each pattern has the same local density for Blue and Green but increasing local density of the Red from Scenario (i) – (iv), as illustrated in the zoomed-in region (Top-left). The resolved TC triplets, where the circle sizes reflect the relative densities of Red from each scenario, are displayed on bottom right. (**b**) Relative local densities of Red from Scenario (i) to (iv) in (a), calculated by TC analysis. Each data point represents result from 1 simulation. 10 replicas of simulation were performed for each scenario. Black horizontal line and box height indicate mean±SD, *N* = 10.

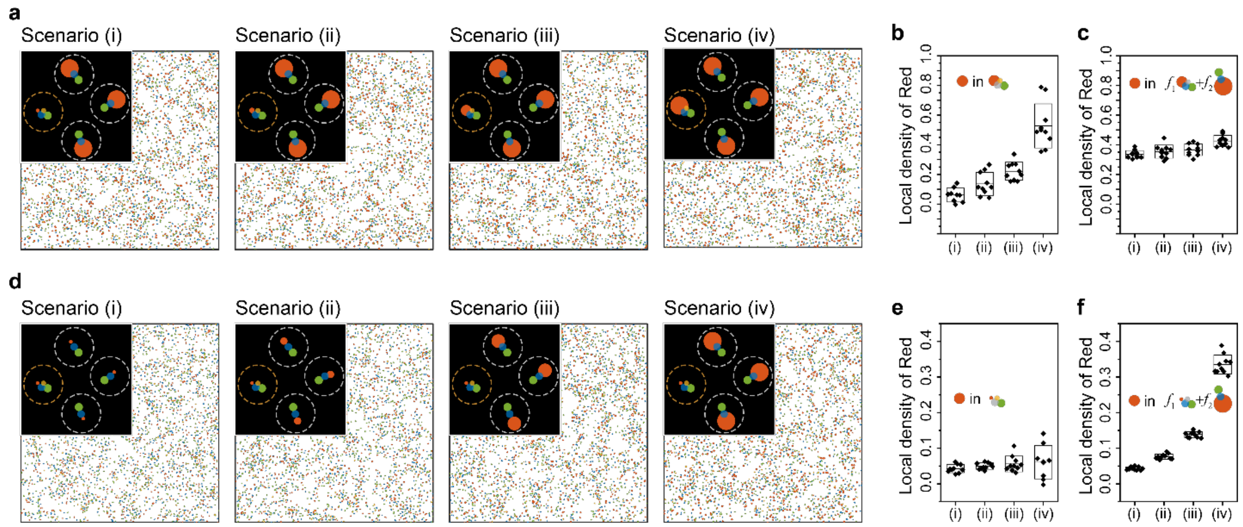

Supplementary Note 3 Figure 3. Validation of the TCF-resolved local density within a triplet pattern via simulations.

(**a**) 1000 YRGB or RGB quadruplets were randomly positioned on the canvas, Each RGB pattern has the same local densities for all individual colors, whereas each YRGB pattern contains the same local density for Yellow, Green, and Blue, but increasing local density of the Red from Scenario (i) – (iv), as illustrated in the zoomed-in region. (**b**) Relative local densities of Red within YRGB pattern from Scenario (i) to (iv) in (a), calculated by TCF. Each data point represents result from 1 simulation. 10 replicas of simulation were performed for each scenario. Box indicates mean±SD, N = 10 replica of simulation. (**c**) Relative local densities of Red within all RGB pattern from Scenario (i) to (iv) in (a), calculated by TC analysis. Each data point represents result from 1 simulation. 10 replicas of simulation were performed for each scenario. Black horizontal line and box height indicate mean±SD, N = 10. (**d**) 1000 YRGB or RGB quadruplets were randomly positioned on the canvas, Each YRGB pattern has the same local densities for all individual colors, whereas each RGB pattern contains the same local density for Green, and Blue, but increasing local density of the Red from Scenario (i) – (iv), as illustrated in the zoomed-in region. (**e**) Relative local densities of Red within YRGB pattern from Scenario (i) to (iv) in (d), calculated by TC analysis. Each data point represents result from 1 simulation. 10 replicas of simulation were performed for each scenario. Box indicates mean±SD, N = 10. (**f**) Relative local densities of Red within all RGB pattern from Scenario (i) to (iv) in (d), calculated by TCF. Each data point represents result from 1 simulation. 10 replicas of simulation were performed for each scenario. Black horizontal line and box height indicate mean±SD, N = 10.

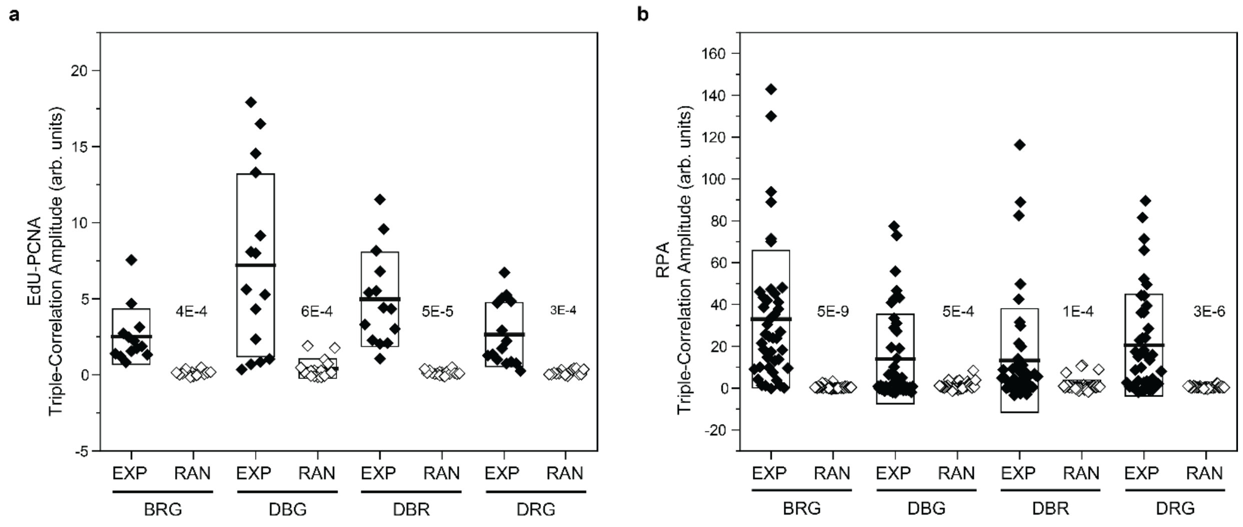

Supplementary Note 3 Figure 4. Validation of the TCF on experimental SMLM data. (a and b) Triple-Correlation analysis showing the magnitudes of triple-stained (B = AF488; R = AF647; G = AF568; D = AF750) colocalization of EdU+PCNA (a) or RPA (b) from experiment (EXP) and randomized (RAN) samples. The significances of EdU+PCNA or RPA self-colocalizations regardless of the labeled fluorophore choice confirms the robustness of the algorithm. Black horizontal line and box height indicate mean±SD, *N* = 15 (a) and 42 (b). Values on graph indicate *p*-values of unpaired two-sample t-tests between EXP and RAN samples.

Supplementary References

1. Raghunandan M, Chaudhury I, Kelich SL, Hanenberg H, Sobeck A. FANCD2, FANCJ and BRCA2 cooperate to promote replication fork recovery independently of the Fanconi Anemia core complex. *Cell Cycle* **14**, 342-353 (2015).

2. De Magis A*, et al.* DNA damage and genome instability by G-quadruplex ligands are mediated by R loops in human cancer cells. *Proc Natl Acad Sci U S A* **116**, 816-825 (2019).

3. Henderson A*, et al.* Detection of G-quadruplex DNA in mammalian cells. *Nucleic Acids Res* **42**, 860-869 (2014).

4. Biffi G, Tannahill D, McCafferty J, Balasubramanian S. Quantitative visualization of DNA G-quadruplex structures in human cells. *Nat Chem* **5**, 182-186 (2013).

5. Biffi G, Di Antonio M, Tannahill D, Balasubramanian S. Visualization and selective chemical targeting of RNA G-quadruplex structures in the cytoplasm of human cells. *Nat Chem* **6**, 75-80 (2014).

6. Jain A, Liu R, Xiang YK, Ha T. Single-molecule pull-down for studying protein interactions. *Nat Protoc* **7**, 445-452 (2012).

7. Coltharp C, Yang X, Xiao J. Quantitative analysis of single-molecule superresolution images. *Curr Opin Struct Biol* **28**, 112-121 (2014).

8. Nicovich PR, Owen DM, Gaus K. Turning single-molecule localization microscopy into a quantitative bioanalytical tool. *Nat Protoc* **12**, 453-460 (2017).

9. Puig Lombardi E, Holmes A, Verga D, Teulade-Fichou MP, Nicolas A, Londono-Vallejo A. Thermodynamically stable and genetically unstable G-quadruplexes are depleted in genomes across species. *Nucleic Acids Res* **47**, 6098-6113 (2019).

10. Chambers VS, Marsico G, Boutell JM, Di Antonio M, Smith GP, Balasubramanian S. High-throughput sequencing of DNA G-quadruplex structures in the human genome. *Nat Biotechnol* **33**, 877-881 (2015).

11. Piazza A*, et al.* Short loop length and high thermal stability determine genomic instability induced by G-quadruplex-forming minisatellites. *EMBO J* **34**, 1718-1734 (2015).

12. Mechali M. Eukaryotic DNA replication origins: many choices for appropriate answers. *Nat Rev Mol Cell Biol* **11**, 728-738 (2010).

13. Hansel-Hertsch R*, et al.* G-quadruplex structures mark human regulatory chromatin. *Nat Genet* **48**, 1267-1272 (2016).

14. Di Antonio M*, et al.* Single-molecule visualization of DNA G-quadruplex formation in live cells. *Nat Chem* **12**, 832-837 (2020).

15. Yin Y, Rothenberg E. Probing the Spatial Organization of Molecular Complexes Using Triple-Pair-Correlation. *Sci Rep* **6**, 30819 (2016).

16. Yin Y, Lee WTC, Rothenberg E. Ultrafast data mining of molecular assemblies in multiplexed high-density super-resolution images. *Nat Commun* **10**, 119 (2019).

17. Rust MJ, Bates M, Zhuang X. Sub-diffraction-limit imaging by stochastic optical reconstruction microscopy (STORM). *Nat Methods* **3**, 793-795 (2006).

18. Betzig E*, et al.* Imaging intracellular fluorescent proteins at nanometer resolution. *Science* **313**, 1642-1645 (2006).

19. Chen YH*, et al.* ATR-mediated phosphorylation of FANCI regulates dormant origin firing in response to replication stress. *Mol Cell* **58**, 323-338 (2015).
