# Supplementary material for: Single-molecule imaging reveals replication fork coupled formation of G-quadruplex structures hinders local replication stress signaling
Source: Nat Commun. 2021 May 5;12:2525. doi: 10.1038/s41467-021-22830-9 (PMC8099879; doi:10.1038/s41467-021-22830-9)
Supplement: Supplementary file 3 — Reporting Summary [file 41467_2021_22830_MOESM3_ESM.pdf]

nature research

Corresponding author(s): Eli Rothenberg

Last updated by author(s): Mar 12, 2021

Reporting Summary

Nature Research wishes to improve the reproducibility of the work that we publish. This form provides structure for consistency and transparency in reporting. For further information on Nature Research policies, see our Editorial Policies and the Editorial Policy Checklist.

Statistics

For all statistical analyses, confirm that the following items are present in the figure legend, table legend, main text, or Methods section.

n/a	Confirmed	
☐	☒	The exact sample size (*n*) for each experimental group/condition, given as a discrete number and unit of measurement
☐	☒	A statement on whether measurements were taken from distinct samples or whether the same sample was measured repeatedly
☐	☒	The statistical test(s) used AND whether they are one- or two-sided<br>*Only common tests should be described solely by name; describe more complex techniques in the Methods section.*
☒	☐	A description of all covariates tested
☒	☐	A description of any assumptions or corrections, such as tests of normality and adjustment for multiple comparisons
☐	☒	A full description of the statistical parameters including central tendency (e.g. means) or other basic estimates (e.g. regression coefficient) AND variation (e.g. standard deviation) or associated estimates of uncertainty (e.g. confidence intervals)
☐	☒	For null hypothesis testing, the test statistic (e.g. *F*, *t*, *r*) with confidence intervals, effect sizes, degrees of freedom and *P* value noted<br>*Give P values as exact values whenever suitable.*
☒	☐	For Bayesian analysis, information on the choice of priors and Markov chain Monte Carlo settings
☒	☐	For hierarchical and complex designs, identification of the appropriate level for tests and full reporting of outcomes
☒	☐	Estimates of effect sizes (e.g. Cohen's *d*, Pearson's *r*), indicating how they were calculated

Our web collection on statistics for biologists contains articles on many of the points above.

Software and code

Policy information about availability of computer code

Data collection	Super-resolution microscopy data were acquired using Micro-Manger (v 1.4).<br>Simulation data were generated using Matlab (v2017a).<br>smFRET data were collected using Andor software (Andor iXon3).<br>Please see Methods section for detailed data acquisition description.
Data analysis	Super-resolution image reconstruction were performed through C++ (via Intel Core i7 7800X) and CUDA8.0 (via NVIDIA GTX 1060) using the Maximum Likelihood Estimation (MLE) algorithm.<br>ebFRET (doi:10.1016/j.bpj.2013.12.055) was used to analyze smFRET data.<br>Data presentation/graphing were performed mainly through Matlab (v2017b) and Originlab(2018).<br>Please see Methods section for detailed data analysis description.<br><br>Codes for Auto- and Triple-Correlation algorithms, as well as a testing demo (with simulation codes) are available at https://github.com/yiny02/direct-Triple-Correlation-Algorithm. The code is for Research and Educational Purposes for Non-Profit Academic and/or Research Institutions.

For manuscripts utilizing custom algorithms or software that are central to the research but not yet described in published literature, software must be made available to editors and reviewers. We strongly encourage code deposition in a community repository (e.g. GitHub). See the Nature Research guidelines for submitting code & software for further information.

Data

Policy information about availability of data

All manuscripts must include a data availability statement. This statement should provide the following information, where applicable:

- Accession codes, unique identifiers, or web links for publicly available datasets
- A list of figures that have associated raw data
- A description of any restrictions on data availability

All imaging and single-molecule data constitute a sizable dataset (>10TB) that cannot be reasonably maintained online. Raw data will be made available by the corresponding author upon request. The full scan western blots (Supplementary Fig. 5a) are provided in the Source Data file.

Field-specific reporting

Please select the one below that is the best fit for your research. If you are not sure, read the appropriate sections before making your selection.

☒ Life sciences ☐ Behavioural & social sciences ☐ Ecological, evolutionary & environmental sciences

For a reference copy of the document with all sections, see nature.com/documents/nr-reporting-summary-flat.pdf

Life sciences study design

All studies must disclose on these points even when the disclosure is negative.

Sample size	For all experiments sample size was not predetermined, as much data as possible was collected depending on the nature of the experiments or in order to perform proper statistical analysis.
Data exclusions	For all imaging experiments, EDU- and/or PCNA-positive (as pre-established S-phase markers) nuclei were selected for analysis, which were then analyzed equally. Details of nucleus selection were described in the manuscript.<br>For smFRET experiments, single-molecule trajectories were truncated to exclude photobleaching events.<br>No other data exclusion were performed.
Replication	All SR experiments were performed at least in triplicate with >60 sample size, as listed in Table S1 in manuscript.<br>smFRET experiments were performed in replicate with >100 sample size.<br>Western blotting experiment was performed in replicate.<br>IF experiment was performed in replicate.<br>SimPull experiment was performed in triplicate.<br>We followed the same protocols to generate replicates for each of our experiments, and the analysis of the data were reliably reproduced.
Randomization	For all imaging experiments, nuclei on coverslips were randomly selected for imaging.<br>For all single-molecule experiments, illuminated molecules were randomly selected for imaging.<br>For western blot experiment, no randomization is required as a proper internal control is present.
Blinding	For all imaging experiments, blinding was not possible as experimental conditions were evident from the image data. Image processing and analysis were done using computational pipelines that were applied equally to all conditions and replicates, therefore do not require blinding.

Reporting for specific materials, systems and methods

We require information from authors about some types of materials, experimental systems and methods used in many studies. Here, indicate whether each material, system or method listed is relevant to your study. If you are not sure if a list item applies to your research, read the appropriate section before selecting a response.

Materials & experimental systems

n/a	Involved in the study	
☐	☒	Antibodies
☐	☒	Eukaryotic cell lines
☒	☐	Palaeontology and archaeology
☒	☐	Animals and other organisms
☒	☐	Human research participants
☒	☐	Clinical data
☒	☐	Dual use research of concern

Methods

n/a	Involved in the study	
☒	☐	ChIP-seq
☒	☐	Flow cytometry
☒	☐	MRI-based neuroimaging

Antibodies

Antibodies used	DNA G4 (1H6), Mouse, Millipore MABE1126

Antibodies used

DNA G4 (1H6), Mouse, Absolute Antibody Ab00389-1.1
DNA G4 (1H6), Rabbit, Absolute Antibody Ab00389-23.0
DNA G4 (BG4), FLAG-tag, Millipore MABE917
MCM6, Rabbit, AF568, Abcam ab211916
PCNA, Mouse, SCBT sc56
γH2AX, Mouse, AF647, Millipore 05-636-AF647
RPA1, Rabbit, AF647, Abcam ab19924
Rabbit IgG AF 750, Invitrogen A21039
Mouse IgG AF 750, Invitrogen A21037
Rabbit IgG AF 647, Invitrogen A21246
Mouse IgG AF 647, Invitrogen A21235
Rabbit IgG AF 568, Invitrogen A11036
Mouse IgG AF 568, Invitrogen A11031
Mouse IgG AF 488, Invitrogen A11029
Rabbit IgG Biotin Abcam Ab6720
RPA2, Rabbit, Bethyl A300-244A
pRPA2 S4/S8, Rabbit, Bethyl A700-009
pRPA2 S33, Rabbit, Bethyl A300-246A
FANCJ, Rabbit, Bethyl A300-561A
MCM5, Rabbit, Bethyl A300-195A
PCNA, Mouse, Abcam Ab29 (PC10)

Validation

All antibodies are from commercially available. Below are links to these products.

DNA G4 (1H6), Mouse, Millipore https://www.emdmillipore.com/US/en/product/Anti-DNA-G-quadruplex-G4-Antibody-clone-1H6,MM_NF-MABE1126
DNA G4 (1H6), Mouse, Absolute Antibody https://absoluteantibody.com/product/anti-quadruplex-dna-1h6/Ab00389-1.1_mouse_igg1/standard/
DNA G4 (1H6), Rabbit, Absolute Antibody https://absoluteantibody.com/product/anti-quadruplex-dna-1h6/Ab00389-23.0_rabbit_igg/
DNA G4 (BG4), FLAG-tag, Millipore https://www.emdmillipore.com/US/en/product/Anti-DNA-G-quadruplex-structures-Antibody-clone-BG4,MM_NF-MABE917
MCM6, Rabbit, AF568, Abcam https://www.abcam.com/mcm6-antibody-epr17686-alexa-fluor-568-ab211916.html
PCNA, Mouse, SCBT https://www.scbt.com/p/pcna-antibody-pc10?gclid=Cj0KCQiAs5eCBhCBARIsAEhk4r4gJzZtFo8GBXSC3HKujdeeWevs47de4ll_3yTfKXjHQP4LCEh9noMaAmbNEALw_wcB
γH2AX, Mouse, AF647, Millipore https://www.emdmillipore.com/US/en/product/Anti-phospho-Histone-H2A.X-Ser139-Antibody-clone-JBW301-Alexa-Fluor-647,MM_NF-05-636-AF647?ReferrerURL=https%3A%2F%2Fwww.google.com%2F
RPA1, Rabbit, AF647, Abcam https://www.abcam.com/alexa-fluor-647-rpa70-antibody-epr3472-ab199240.html
Rabbit IgG AF 750, Invitrogen https://www.thermofisher.com/antibody/product/Goat-anti-Rabbit-IgG-H-L-Cross-Adsorbed-Secondary-Antibody-Polyclonal/A-21039
Mouse IgG AF 750, Invitrogen https://www.thermofisher.com/antibody/product/Goat-anti-Mouse-IgG-H-L-Cross-Adsorbed-Secondary-Antibody-Polyclonal/A-21037
Rabbit IgG AF 647, Invitrogen https://www.thermofisher.com/antibody/product/Goat-anti-Rabbit-IgG-H-L-Cross-Adsorbed-Secondary-Antibody-Polyclonal/A-21246
Mouse IgG AF 647, Invitrogen https://www.thermofisher.com/antibody/product/Goat-anti-Mouse-IgG-H-L-Cross-Adsorbed-Secondary-Antibody-Polyclonal/A-21235
Rabbit IgG AF 568, Invitrogen https://www.thermofisher.com/antibody/product/Goat-anti-Rabbit-IgG-H-L-Highly-Cross-Adsorbed-Secondary-Antibody-Polyclonal/A-11036
Mouse IgG AF 568, Invitrogen https://www.thermofisher.com/antibody/product/Goat-anti-Mouse-IgG-H-L-Highly-Cross-Adsorbed-Secondary-Antibody-Polyclonal/A-11031
Mouse IgG AF 488, Invitrogen https://www.thermofisher.com/antibody/product/Goat-anti-Mouse-IgG-H-L-Highly-Cross-Adsorbed-Secondary-Antibody-Polyclonal/A-11029
Rabbit IgG Biotin Abcam https://www.abcam.com/goat-rabbit-igg-hl-biotin-ab6720.html
RPA2, Rabbit, Bethyl https://www.bethyl.com/product/A300-244A/RPA32+Antibody
pRPA2 S4/S8, Rabbit, Bethyl https://www.bethyl.com/product/A700-009
pRPA2 S33, Rabbit, Bethyl https://www.bethyl.com/product/A300-246A/Phospho+RPA32+(S33)+Antibody
FANCJ, Rabbit, Bethyl https://www.bethyl.com/product/A300-561A/BRIP1+BACH1+Antibody
MCM5, Rabbit, Bethyl https://www.bethyl.com/product/A300-195A/MCM5+Antibody
PCNA, Mouse, Abcam https://www.abcam.com/pcna-antibody-pc10-ab29.html

Eukaryotic cell lines

Policy information about cell lines

Cell line source(s)

U2OS cell lines were obtained from ATCC.
HeLa cells were originally provided by Prof. Stephen Taylor (University of Manchester, UK, DOI: 10.1083/jcb.200712028). The cell line was obtained with the authorization to use it for academic research purposes.

Authentication

No cell line authentication was performed.

Mycoplasma contamination

No Mycoplasma contamination test was performed.

Commonly misidentified lines<br>(See ICLAC register)	No commonly misidentified lines were used.
